# Supplementary material for: Aquatic insect community structure revealed by eDNA metabarcoding derives indices for environmental assessment
Source: PeerJ. 2020 Jun 11;8:e9176. doi: 10.7717/peerj.9176 (PMC7293852; doi:10.7717/peerj.9176)
Supplement: Supplemental Information 8 [file peerj-08-9176-s008.docx]

**Text S1.** Determine sequence identity threshold for assignment of aquatic insects using the CO1 region of mitochondrial DNA.

Methods:

For assignment of the taxonomy of aquatic insects, we surveyed sequence identity of Cytochrome Oxidase subunit 1 (CO1) region of mitochondrial DNA at the order, family, genus and species level. We focused on three aquatic insect orders, Ephemeroptera, Plecoptera and Trichoptera (E/P/T) because they are popular in aquatic ecosystem and important to assess river environment. For analysis, nine species were selected from orders EPT since they frequently appeared in the study area. Sequence data were obtained from NCBI database in August 2018. The obtained data were used to create a database using the Arb software (The ARB Project) to extract suitable sequence data (i.e., sequence length >300 bp and published in any journal), and randomly selected sequences for each species were used for calculation. Subsequently, the data were aligned using Clustal X and the sequence identity among the species, genus, family and order level were calculated using “dnadist” application of Philips ver. 3.67.

Results:

Intra-species sequence identity was extremely high (median ± SE =0.991 ± 0.006) meanwhile intra-genus sequence identity was drastically reduced (median ± SE =0.854 ± 0.019) (S2 Table). Intra-family and intra-order sequence identities were 0.831 ± 0.019 and 0.779 ± 0.007, respectively. This result was consistent with Wakimura et al. (2016) who investigated that genetic similarities among several species of Ephemeroptera corrected from Japan and Taiwan, said the CO1 region was strongly preserved the sequence among species, but highly mutated among higher taxonomic level. The previous study used the sequence identity of 90% for the family level assignment (2). But the based on the analysis (Table), 90% sequence identity seems high for family level assignment. Setting higher threshold reduce the risk of false-positive but increase the rate of false-negative. Therefore, in this study, sequence identity of 85%, corresponding to intra-genus sequence identity, was used for taxonomic assignment at the family level.

Table:

References:

1. Fernández S, Rodríguez S, Martínez JL, Borrell YJ, Ardura A, García-Vázquez E. Evaluating freshwater macroinvertebrates from eDNA metabarcoding: A river Nalón case study. PLoS ONE [Internet]. 2018 Aug 8 [cited 2018 Sep 7];13(8). Available from: https://www.ncbi.nlm.nih.gov/pmc/articles/PMC6082553/

2. Wakimura K, Takemon Y, Takayanagi A, Ishiwata S, Watanabe K, Tanida K, et al. Characterization of genes for histone H3, 18S rRNA, and cytochrome oxidase subunit I of East Asian mayflies (Ephemeroptera). :26.
