# Supplementary material for: Aquatic insect community structure revealed by eDNA metabarcoding derives indices for environmental assessment
Source: PeerJ. 2020 Jun 11;8:e9176. doi: 10.7717/peerj.9176 (PMC7293852; doi:10.7717/peerj.9176)
Supplement: Supplemental Information 9 [file peerj-08-9176-s009.docx]

**Text S2. How to calculate EPT and Diptera index for the aquatic insect community revealed by conventional and eDNA methods**

- For conventional Surber net survey data

Metrics based on abundance using individual counts;

$$\%EPT=\frac{Total EPT taxa individuals}{Total individuals of the samples}\times100\%$$

$$\%Diptera=\frac{Total Diptera taxa individuals}{Total individuals of the sample}\times100\%$$

$$\%Chironomidae=\frac{Total Chironomidae taxa individuals}{Total individuals of the sample}\times100\%$$

- For eDNA data analyzed at genus/family-level identification

Metrics based on OTU richness (the number of OTUs);

$$\%EPT=\frac{Total EPT OTU richness}{Total OTU richness}\times100\%$$

$$\%Diptera=\frac{Total Diptera OTU richness}{Total OTU richness}\times100\%$$

$$\%Chironomidae=\frac{Total Diptera OTU richness}{Total OTU richness}\times100\%$$
